# FOCUSED–Short-Term Wind Speed Forecast Correction Algorithm Based on Successive NWP Forecasts for Use in Traffic Control Decision Support Systems

**DOI:** 10.3390/s21103405

**Published:** 2021-05-13

**Authors:** Zdravko Kunić, Bernard Ženko, Biljana Mileva Boshkoska

**Affiliations:** 1 Faculty of Information Studies in Novo Mesto, 8000 Novo Mesto, Slovenia; biljana.mileva@fis.unm.si; 2 Department for Information Systems and Business Analytics, Algebra University College, 10000 Zagreb, Croatia; 3 “Jožef Stefan” Institute, Jamova Cesta 39, 1000 Ljubljana, Slovenia; bernard.zenko@ijs.si

**Keywords:** traffic management, wind speed prediction, forecast correction, neural networks, successive forecasts

## Abstract

In this paper, we propose a new algorithm, called FOCUSED (FOrecast Correction Using Successive prEDictions), for forecast correction of short-term wind speed predictions. We developed FOCUSED with the aim of improving the forecast of bora gusts, which frequently result in high-speed wind situations dangerous for traffic. The motivation arises from occasionally ambiguous results of the currently deployed decision support system, which aids traffic management in strong and gusty wind conditions at the coast of Croatia. The proposed correction algorithm uses characteristics of numerical weather prediction models to iteratively forecast the wind speed multiple times for the same future window. We use these iterative predictions as input features of the FOCUSED algorithm and get the corrected predictions as the output. We compared the proposed algorithm with artificial neural networks, random forests, support vector machines, and linear regression to demonstrate the superiority of the algorithm’s performance on a data set comprising five years of real data measurements at the Croatian bridge “Krk” and complementary historical forecasts by ALADIN (Aire Limitée Adaptation dynamique Développement InterNational) numerical weather prediction model.

## 1. Introduction

Bora (Bura in Croatian) is a cold, strong and gusty wind that blows along the Eastern Adriatic coast and islands. Each winter, several damaging bora storms hit the coastal region of Croatia, strongly affecting sea, air and road transport safety, and life in general [1]. In particular, bora can have a severe negative impact on road traffic security [2]. In critical situations, road managers use wind forecasting models as an aid in making decisions to open or close a road for traffic or to define new speed limits. Three parameters define a critical wind situation in the context of traffic control in the Eastern Adriatic coast: wind direction, 10-min mean wind speed and 10-min maximum wind speed. For each location, these parameters define critical wind situations that vary depending on a specific terrain configuration and pavement condition (dry/wet/icy) [2].

According to the time-horizon classification [3], in the process of wind modelling and wind forecast correction, we distinguish three different forecasting time scales: immediate short-term (8 h ahead), short-term (a day ahead) and long-term (multiple days ahead) forecasts. The ALADIN [4] forecast is used by an Anemo-Alarm [5] traffic control decision support system (DSS) twice a day. Due to long time intervals between two consecutive forecasts, traffic managers occasionally are presented with contradictory measurements (e.g., current wind speed) and forecasts from ALADIN. Hence, road managers have to deal with boundary cases in which they have to decide about decreasing maximum allowed speed limits or even closing a road based on inconsistent or ambiguous forecasts and measurements. The most evident problem of the existing decision support system is its inability to suggest a right decision when there are significant differences between forecasted and measured wind speed trends, and especially when the trends are opposite. In such cases, road managers may select a decision which can lead to potentially disastrous consequences. In particular, the decision to close a road due to strong wind may lead to substantial loss of drivers’ time, as well as a financial loss for drivers and companies, and consequently, the country’s economy. On the other hand, a decision of leaving a road open may lead to significant material damage or even more catastrophic scenarios, including life losses. It is, therefore, important to provide the best possible wind forecasts to support decision makers, and in particular, it is important to provide them with wind forecasts between two consecutive ALADIN forecasts.

To address this problem, we propose a correction algorithm, called FOCUSED, that uses existing forecasts from ALADIN as inputs to an ANN (Artificial Neural Network) and outputs corrected predictions. The proposed algorithm aims to improve the immediate-short-term and short-term (1 to 12 h in advance) wind forecast accuracy during the period when a newer ALADIN forecast is not available and supports road managers in the process of choosing the most appropriate action based on timely and relevant information. In this research we show that the latest successive forecast is not always the best forecast and that it is possible to improve the wind speed forecast accuracy using only consecutive forecasts of the existing physical forecast models (i.e., ALADIN), without any additional mathematical or physical input features.

FOCUSED can be used with any data-mining model, therefore we compared its usage with artificial neural networks, random forests, support vector machines, and linear regression to demonstrate the superiority of the algorithm’s performance on a data set comprising five years of real data measurements at the Croatian bridge “Krk” and complementary historical forecasts by ALADIN numerical weather prediction model.

The motivation for the proposed algorithm comes from the challenges posed by the existing forecasting system at the “Krk” bridge in Croatia. In particular, our objective is to improve prediction of short-burst critical high-speed wind situations that are dangerous for traffic. Our solution can easily be integrated into the existing decision support system and is computationally inexpensive. However, usability of the proposed method is not limited to the above scenario and can be also used for improving forecasts in general, for example, for forecasting wind power plant production.

## 2. Related Work

In meteorology, the improvement of the forecasts is performed by employing post-processing methods using local measurements and weather prediction model outputs [6]. Model improvements using correction algorithms represent an acceptable option because they improve the inaccurate representation of atmospheric state by NWP (Numerical Weather Prediction) models and decrease observation errors [7]. In general, a correction algorithm feeds the input features into a “correction” model that outputs corrected original predictions. Input features can include data not utilized by the original NWP model, for instance, orography roughness optimization (considering more detailed topographic relief), historical data, real-time measured data and calculated errors. The correction model itself can be a result of a broad spectrum of data-mining and post-processing methods, as we describe below. It can also be based on the latest results obtained by the NWP models or unused features in the original NWP models. Many correction algorithms have their origins in Kalman filtering [8,9,10], neural networks [11] and their combination [12], as well as other statistical methods which combine different post-processing methods to reduce errors of physical models [10].

Kalman filtering is one of the popular post-processing methods [8,9,10]. According to [13], when dealing with limited-area atmospheric models with different options and capabilities of horizontal resolution, it leads to the elimination of possible systematic errors, even in lower resolution cases, contributing further to significant reduction of the required CPU time. In [14], the error forecast models are based on a support vector machine or extreme learning machine. In [15], authors propose wind power forecast correction by subtracting the biased mean from the wind power forecast error.

A variational method [7] for correcting non-systematic NWP forecast errors is based on previous numerical forecasts assuming that the error is linearly dependent on some combination of the forecast fields. Using the Single Value Decomposition (SVD) of the covariance matrix between the forecast and forecasting they obtain the inverse mapping from flow space to the error space during the training period. To avoid the difficulty in solving the inverse matrix, they reduced the background covariance matrix to a simple diagonal matrix.

Errors of wind speed forecasts can also be reduced by searching for an optimal combination of post-processing methods, as described in [10] where adaptive approaches to post-processing wind speed forecasts are discussed and compared using automatic methods for combining forecast streams.

In the syntheses-correcting forecasting model [16], the wind speed forecasting bias correction method is based on the Empirical Orthogonal Function (EOF) and regression analysis.

In [6], the correction models use polynomial neural networks for modelling real complex systems. This method also revises forecasts according to the corrective function that depends on real observations.

Combined forecasting methods, proposed in [14], investigate the correlation relationships of forecast errors of the autoregressive model, the persistence method and the support vector machine model in various forecasting modes. Authors proposed a strategy for selecting the input variables and defined the range of input variables according to the results of the correlation analysis.

In [17], the authors aim to improve the performance of the real-time decomposition-based forecasting method after they uncover the factors attributed to its unsatisfactory performance. They decomposed the raw wind speed time series into a different subseries. To reduce the disturbance of illusive components, they used (a) kernel density estimation-based Kullback-Leibler divergence and (b) energy measure. Finally, the hybrid of least squares support vector machine and generalized autoregressive conditionally heteroscedastic model is introduced to correct the resulting error component if its inherent correlation and heteroscedasticity cannot be neglected [17].

Related to the problem of short-time wind speed forecast correction with the aim of improving decision support systems for traffic control in dangerous wind situation is also the problem of wind farm power prediction. For example, in the study [18] about wind farm NWP wind speed correction methods, measured time series were decomposed into different bands by wavelet multi-resolution analysis. Correction premise was verified using the Pearson product-moment correlation coefficient, and then the linear correction method was used to correct the low-frequency stationary NWP wind speed.

We started the research with the hypothesis that the last NWP forecast is not always the most accurate existing forecast. None of the abovementioned research and correction algorithms take into consideration previous forecasts for the same future time period, and they also do not differentiate continuous wind speed/direction measurements from critical wind speed/direction situations for traffic.

The most related algorithm to FOCUSED is a forecast error correction method in numerical weather prediction using recent multiple-time evolution data [19]. It also falls into the category of those who act as if the last forecast is the best forecast. It compares the first part of the forecasted values with actual measured values to find a function that describes the forecast error and uses this function to the rest of the forecast to correct the errors. The model error is expressed as a Lagrange interpolation polynomial, while the coefficients of the polynomial are determined by past model performance. Both algorithms, FOCUSED and recent multiple-time evolution data divide forecasted time series data into two parts: past and future forecasted data, with respect to the observation point (which is in fact the present time). Both algorithms need some initial time after receiving new forecasts to calculate initial errors. The differences start with the scope and evaluation data set—the scope of the recent multiple-time evolution algorithm is general NWP correction based on vectors of multiple various meteorological dimensions and evaluation is based on an artificially created data set, while our scope is only wind speed correction without using other variables, and evaluation is based on a real data set. To generate corrected time series with the best possible congruence between forecasted and measured data recent multiple-time evolution searches for an error function between two time series (one forecast and one measurement) using Lagrangean interpolation polynomial, we combine multiple time series (multiple forecasts and one measurement) using ML recent multiple-time evolution use flow of last *n* samples of vectors (of one recent forecast), and we use groups of samples for the same forecasted period/interval (multiple time series data of multiple recent forecasts).

Therefore, we propose a novel algorithm in this paper that differs from existing algorithms by taking into consideration multiple successive forecasts instead of only the last one and a single bora duration as the time horizon for training the data set instead of historical data for the observed location.

## 3. Materials and Methods

### 3.1. Forecasting Setup

**Figure 1:**
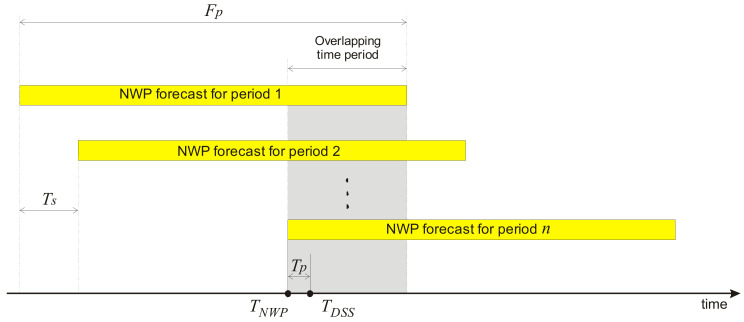
A group of successive forecasts.

The FOCUSED algorithm is a correction algorithm which uses previous forecasts from the NWP model as its inputs (Figure 1). Forecasts that share particular outcome periods are grouped together. Generally, any NWP model whose outputs are to be used in FOCUSED needs to calculate a forecast for the exact number of hours ahead $F_{p}$ (forecast period), to be scheduled to run in regular time intervals $T_{s}$ (time between two consecutive NWP model runs), and it may spend some processing time $T_{p}$ (time of processing) to acquire new data and make the output of a physical model available to the DSS. $T_{NWP}$ denote the point in time when NWP has created the forecasts in a successive group, and $T_{DSS}$ denotes the point in time when the forecast group becomes available to DSS. The data set used in this research consists of two parts: hourly wind speed/direction forecasts for 72 h periods calculated every 12 h, and actual wind speed/direction data measured every 10 min.

Ideally, the correction algorithm should get all forecasts at time $T_{NWP}$. However, a delay of $T_{p}$ can occur between the end time of forecast modelling and the time when DSS gets the data, which is a result of a data synchronization process between the modelling system and decision support system. It may last from several seconds to several hours, depending on the infrastructural and business relationships between the NWP forecast provider and decision support system user.

Knowing the forecast period $F_{p}$, the time gap $T_{p}$ and the time $T_{s}$ between two consecutive runs of NWP, we can calculate the number of overlapping forecasts, which we denote as a number of relevant previous forecasts ($N_{pf}$). We calculate the number of relevant previous forecasts as an input parameter to the proposed algorithm as:

(1) $$N_{pf}=A\frac{\left(F_{p}-T_{p}\right)}{T_{s}}$$

Ideally, the $N_{pf}$ should be equal to the number of consecutive forecasts in order to use all whole available previous forecasts, however due to $T_{p}$, this number may decline because we will lose an hour of the oldest forecast for each hour of $T_{p}$.

### 3.2. The FOCUSED Algorithm

**Figure 2:**
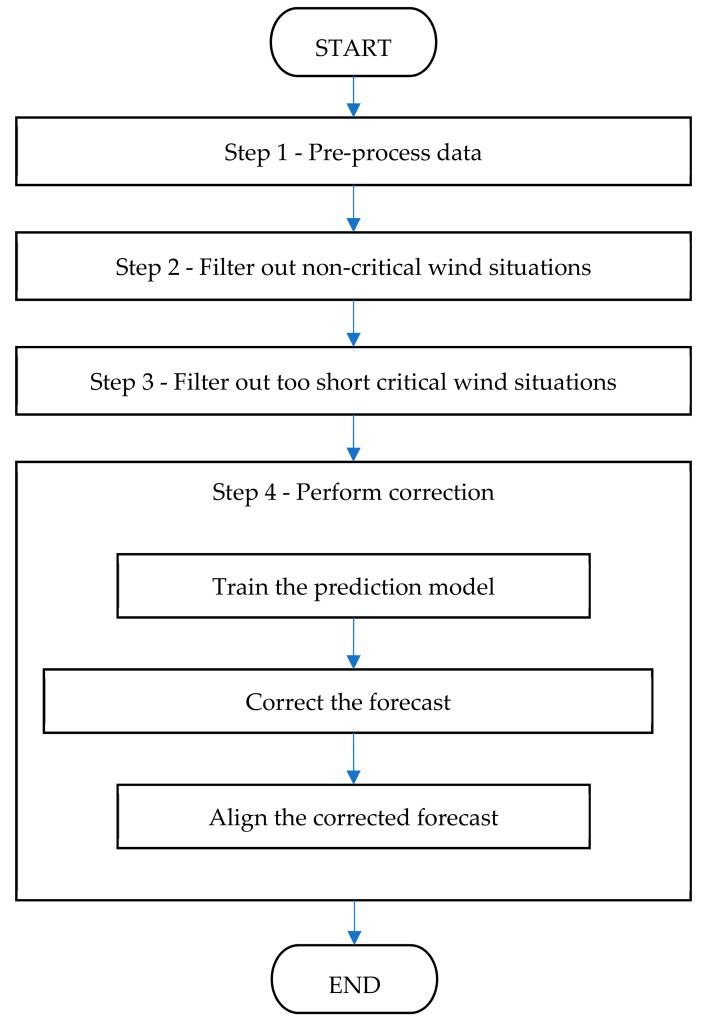
Steps of the FOCUSED algorithm.

The FOCUSED algorithm consists of four main steps and is presented in Figure 2.

We used multiple models to test the performance of the algorithm: Artificial Neural Networks (ANN), Random Forests (RF), Support Vector Machines (SVM) and Linear Regression (LR). Since there is no difference in steps of the algorithm when different data-mining methods are used, we will thoroughly walkthrough using ANN, the model that in this research statistically performed the best.

**Figure 3:**
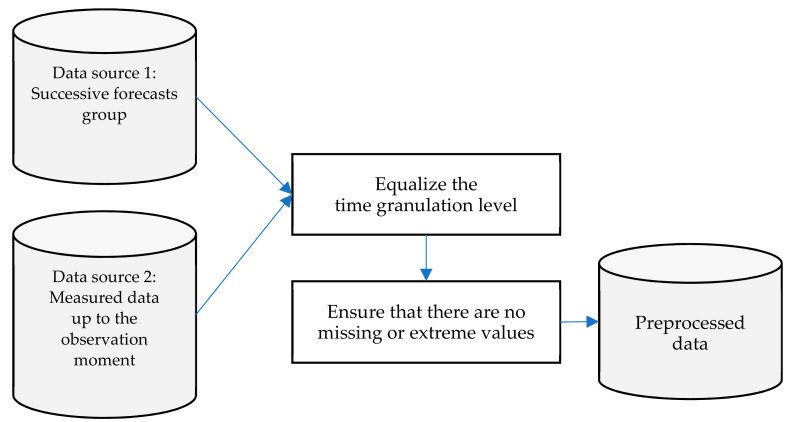
Data pre-processing steps.

The first step is data pre-processing (Figure 3). We used two types of input data: successive overlapping forecasts for a future 12 h period, and actual measured data up to the observation moment. In the pre-processing step, all input data are normalized to the same time granulation level (in our case, 1 h) and it is ensured that there are no missing or extreme values in the data set. Additionally, one should be aware that accuracy of corrected wind speed obtained by running the FOCUSED algorithm is also subject to input data integrity issues. In this research we used validated data sets, but in order to offer a universal guide we should point out the need to address integrity issues as well. They can arise from two main directions: (a) unintentionally unreliable measurements (hardware or software errors or malfunctions) or (b) intentional data disruption–e.g., cyber-attacks related to data integrity that are expected to harm the performances of forecasting systems [20], or false data injection attacks against wind power deterministic and probabilistic forecasting [21].

**Figure 4:**
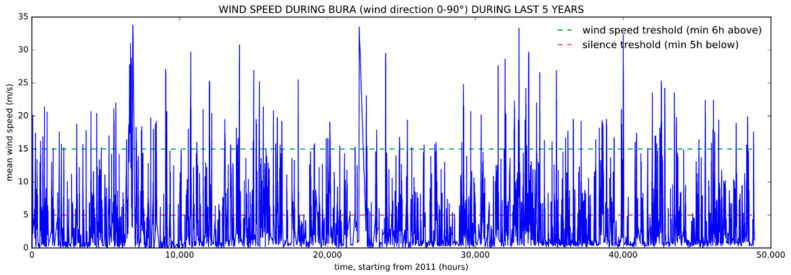
Hourly average wind speed measurements.

The next step is the filtering of non-critical wind situations from the data set. This step is important because the algorithm is intended to support decisions regarding road closure or speed limitations during strong wind situations, hence the need to isolate only such conditions. To distinguish critical from non-critical wind situations, we defined the starting and the ending point of the single bora period. For the purpose of this study which uses continuous data from five consecutive years, we identified a critical bora situation as the wind that blows from any direction between north and east (0–90°), whose average hourly speed remains above wind speed threshold for at least 6 h and lasts until the speed drops below the silence threshold for at least 5 h. In Croatia, a wind speed that is higher than 17 m/s represents one of national weather alert criteria [22]. Therefore, the upper wind speed threshold is set to that speed, and for the same reason the silence threshold is set to 5 m/s, just below the lower boundary during icy road condition periods. In Figure 4, we show an example of an hourly average measurements during the five years with upper and lower boundaries used to identify critical wind situations.

Step 3 of the FOCUSED algorithm comprises filtering out too short critical wind situations. In particular, we decided to exclude critical winds shorter than 24 h from the research, which is a period that we divided to a minimum of 12 h of data for training the model (training data set) and 12 h of data for testing the model’s performance (testing data set), as shown in Table 1. In windy situations which last longer than 24 h, we created different lengths of a training set, while the length of the test data set was always 12 h. In this research, one hour of time series wind data is aggregated and is denoted as one data sample. Each strong wind situation with the length of $n_{t}$ (total number of samples) was repeatedly tested $n_{r}$ (number of algorithm runs) times:

(2) $$n_{r}=n_{t}-\left(24-1\right)=n_{t}-23.$$

For example, if the time interval of Bura is 27 h, resulting in $n_{t}=27$ samples, then $n_{r}=27-\left(24-1\right)=4$ algorithm runs.

In the last algorithm step, Step 4, we performed training of the model for each particular strong wind situation, defined in Step 3, to get the initial prediction FC (Forecast Corrected). We defined the training period as a minimum of 12 h after detecting a strong wind situation and created an input vector of successive wind speed time series.

**Figure 5:**
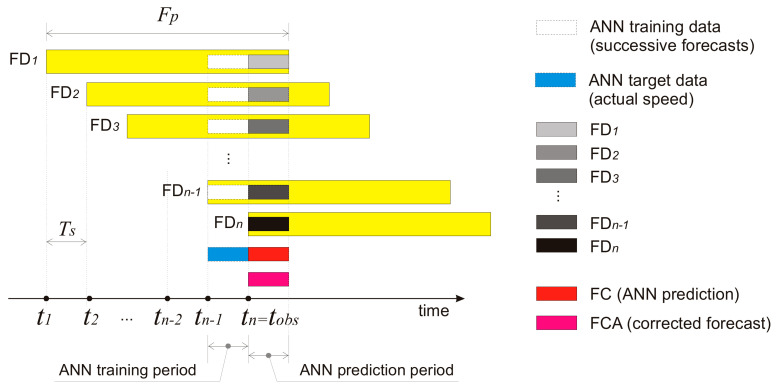
Data sets immediately after receiving a new NWP forecast.

The correction between two consecutive NWP forecasts (Figure 5) is based on a forecast group that starts at *t*_1_ and lasts for *n* 12 h time slots. The oldest forecast FD_1_ (Forecasted Data 1) starts at *t*_1_ and contributes to the model with its fifth (training/test data set) and sixth (evaluation data set) time slots. The next forecast FD_2_ starts at *t*_2_ and contributes to the model with its fourth and fifth time slots, and so on until the last forecast FD_6_ starts at *t*_*n*−1_ and contributes to the model with its first two time slots.

There are *n* consecutive runs of the NWP model, resulting in *n* forecasts (FD_1_–FD_n_) for the respective periods $F_{p}$, denoted in yellow. The white boxes denote the forecasts used as the data-mining model’s input variables and the blue box denotes actual measured wind speed used as a target variable. Gray colors denote successive forecasts predicted by the NWP model for the observed future 12 h period.

**Figure 6:**
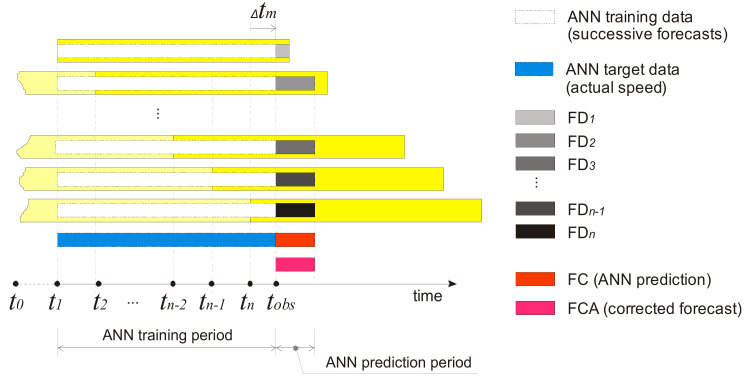
ANN data sets during long critical wind situations.

Consider that Fp is 72 h and $T_{s}$ is 12 h. At the time *t*_1_, the NWP model has created a forecast for the next 72 h, denoted as FD_1_. At the time *t*_2_, the NWP model makes a new forecast for the next 72 h denoted as FD_2_, and so on. During one $F_{p}$ period, the NWP model makes six forecasts whose lengths are equal to the $T_{s}$ period. In application where the proposed algorithm is used as a part of real-time decision support system, the modelling process should repeat each hour, immediately after receiving new measured hourly wind-speed data. When the critical wind situation lasts longer than $F_{p}$, the training set becomes larger than $F_{p}$ (Figure 6). Every new NWP forecast becomes FD_n._ Previous FD_n_ becomes FD_n−1_, and at the end FD_2_ becomes FD_1_, hence the training set could grow indefinitely.

We used a group of forecasts from the time slot between *t*_0+__12h_ (12 h after start of critical wind condition) and *t_obs_* (observation point) as input vectors to train the model. The model’s performance was tested on time-slot data between *t_obs_* and *t_obs+_*_12h_.

To test the algorithm’s performance, the data was split into training and test subsets according to the length of the bora situation and actual observation point. The training subset consisted of 12 h data or more, depending on the length of critical bora situation, calculated with:

(3) $$X_{length}=B_{length}-n,\;\forall n\;:\;T_{s}\;\leq n\leq \left(B_{length}-T_{s}\right)$$

where $X_{length}$ is the length of training subset, $B_{length}$ is the length of the whole observed bora situation, and $T_{s}$ is the time between two consecutive NWP model runs.

**Figure 7:**
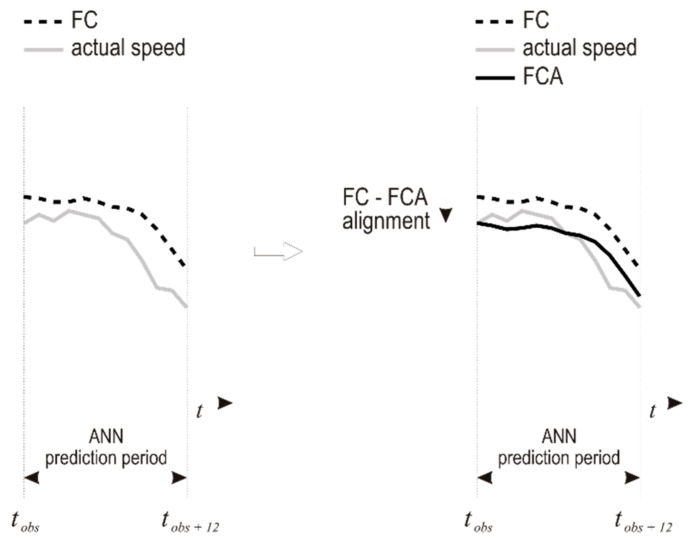
Alignment of corrected forecast to the last measured value.

The final part of the fourth step of the algorithm is vertical shift of the corrected curve, so that first corrected wind speed data point is aligned with the last measured data point (Figure 7). The end result of the proposed algorithm is FCA (FC aligned) which keeps the shape of the FC, with its beginning point aligned to the last measured value.

### 3.3. Training the Model (ANN Example)

**Figure 8:**
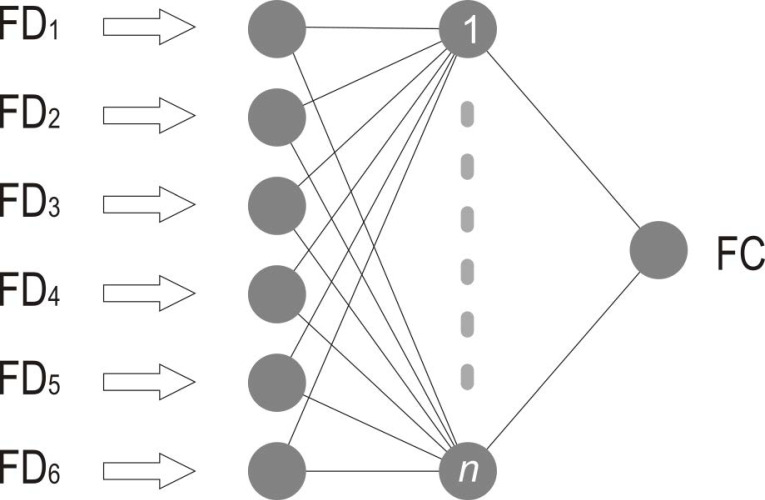
Input vectors of ANN with *n* neurons in hidden layer.

Among available ANN methods, for our correction model we used a MLP (Multi-Layer Perceptron) regressor with three hidden layers of 500 neurons and ReLU (Rectified Linear Unit) activation function. Multi-layer perceptron represents a type of ANN that consists of at least three layers of artificial neurons: input, hidden and output (Figure 8).

**Figure 9:**
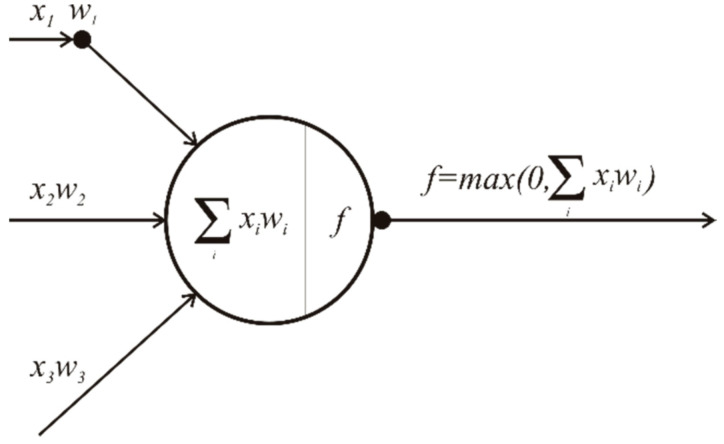
Artificial neuron.

Neurons between input/hidden and hidden/output layers are connected by weighted connections that produce output based on the feature vectors, weights of the connections and non-linear activation function (Figure 9).

The learning process starts with random initialization of weights and calculation of the ANN output based on the first feature vector from the training data set as an input. Given the input values X, weights w, and activation function f, the ANN algorithm calculates the output y of each neuron using [23]:

(4) $$y=f\left(X_{1}w_{1}+X_{2}w_{2}+\ldots +X_{n}w_{n}\right)$$

The activation function f introduces non-linearity to the weighted sum of the neuron’s connections to better fit the real-world data. Some commonly used activation functions are sigmoid, tanh and ReLU [24]:

1. sigmoid

(5) $$f\left(x\right)=\frac{1}{\left(1+e^{-x}\right)}$$

2. tanh

(6) $$f\left(x\right)=2\sigma \left(2x\right)-1$$

3. ReLU

(7) $$f\left(x\right)=\max\left(0,x\right)$$

We used ReLU because of performance superiority and also to avoid the vanishing gradient problem present with sigmoid and tanh functions [25]. Output *o* of each neuron *i* calculated using ReLU activation function is given by:

(8) $$o_{i}=\max\left(0,\;\overset{n}{\underset{k=1}{\sum }}w_{ki}o_{k}\right).$$

The ANN algorithm compares the final output of neural network *o*, for respective feature vector *i*, with the target value *t*(*i*) giving the degree of error *err*:

(9) $$err_{i}=t_{i}-o_{i}$$

Depending on the difference between target and ANN output value, the algorithm updates the weights through a back-propagation process [23] of minimizing the squared error ε in the output of each neuron *i*, which is given by:

(10) $$\varepsilon =\underset{i}{\sum }err_{i}^{2},$$

During the learning process, MLP iteratively adjusts the weights of these connections using the back-propagation process in order to find weights, such that the output value for every input vector in the training set yields the closest value to the target value. The algorithm steers the refinement of the weights by partial derivatives [23] used to decrease the error ε gradually:

(11) $$w_{i}^{\prime }=w_{i}-\eta \frac{\partial \varepsilon_{total}}{\partial w_{i}},$$

where $w_{i}^{\prime }$, is the new weight of the *i*-th neuron, while η represents the learning rate.

The back-propagation process repeats until one of three conditions are met: (a) a predefined number of iterations is achieved, (b) error below a predefined value is minimized, or (c) weights do not change significantly between iterations given a chosen threshold value. After training the MLP, the model is ready to predict the outputs for new, previously unseen data input feature vectors.

## 4. Results

As the FOCUSED algorithm substantially differs from existing wind-speed correction algorithms (described in Section 2: Related Work), we conducted three heterogeneous tests to show its potential. We provide evaluation that brings (a) statistical evaluation that confirms the potential of using previous successive forecasts as input features, (b) comparison with the widely used Autoregressive Moving Average model (ARMA) based on modelling residual errors to correct predictions, and (c) empirical evaluation that shows insights from the traffic manager’s point of view, who is the main stakeholder which will benefit from the use of this algorithm.

### 4.1. Statistical Evaluation of Using Successive Forecasts Instead of the Last Forecast

We tested the algorithm using four data-mining models: ANN, RF, SVM and LR. We compared and evaluated their respective error distributions with Mann-Whitney U Tests [26].

The Mann-Whitney U Test is a nonparametric statistical procedure for comparing two independent, non-related samples. In our case, the last NWP model’s FD_6_ prediction was compared with each of the four data-mining models predictions, respectively. Input samples were created as lists of percentages of forecast/correction errors compared to actual measured wind speed, calculated as:

(12) $$MW_{1}=\frac{FD_{6}-sp_{mean}}{sp_{mean}}\;MW_{2}=\frac{MLM\;–\;sp_{mean}}{sp_{mean}},$$

where $MW_{1}$ and $MW_{2}$ are Mann-Whitney input values, FD_6_ is the last NWP model’s forecast, *MLM* is the machine-learning model’s corrected forecast value, and *sp_mean_* is actual measured wind speed.

Two samples were then combined and sorted in order to determine if the values from the two samples were clustered or randomly mixed. Mann-Whitney U Test statistic for each of the samples was determined by the following formula:

(13) $$U_{i}=n_{1}n_{2}+\frac{n_{i}\left(n_{i}+1\right)}{2}-\sum R_{i}$$

where $U_{i}$ is the test statistic for respective samples, $n_{1}$ and $n_{2}$ are the length of the $MW_{1}$ and $MW_{2}$ samples, and $R_{i}$ represents the sum of ranks for each sample.

The next step was the examination for significance, for which we stated the null and research hypotheses as:

- H_0_ (null hypothesis): There is no tendency for ranks of corrected forecasts based on tested models to be significantly different than ranks of the original NWP (FD_6_).
- H_1_ (research hypothesis): The ranks of corrected forecasts are significantly different than those of the FD_6_.

Our research hypothesis was directional because it indicates the expectation of lower values for the sample related to corrections calculated by FOCUSED algorithm, and higher values for the sample related to NWP FD_6_ predictions. We calculated $U_{1}$ for FD_6_ predictions and $U_{2}$ for respective data-mining corrections.

The existence of clustered groups would indicate that there is significant difference between samples, and a random rank order would mean that there is no difference between samples.

After computing the U statistic, we calculated the *z*-score.

(14) $$z=\frac{U-m_{U}}{\sigma_{U}},$$

where $m_{U}$ is mean deviation of *U* given by:

(15) $$m_{U}=\frac{n_{1}n_{2}}{2},$$

and $\sigma_{U}$ is standard deviation of *U* given by:

(16) $$\sigma_{U}=\sqrt{\frac{n_{1}n_{2}}{12}\left(\left(n+1\right)-\overset{k}{\underset{i=1}{\sum }}\frac{t_{i}^{3}-t_{i}}{n\left(n+1\right)}\right)},$$

where *n* = *n*_1_ + *n*_2_, $t_{i}$ is the number of subjects sharing rank *i*, and *k* is the number of distinct ranks.

The results are shown in Table 2.

Input vectors are expressed as percentage error differences between actual and predicted values. Therefore, lower U-values are considered better.

### 4.2. Statistical Comparison with Autoregressive Moving Average (ARMA) Model

The Autoregression (AR) method models the next step in the sequence as a linear function of the observations at prior time steps. The Moving Average (MA) method models the next step in the sequence as a linear function of the residual errors from a mean process at prior time steps. The Autoregressive Moving Average (ARMA) method models the next step in the sequence as a linear function of the observations and residual errors at prior time steps. It combines both Autoregression (AR) and Moving Average (MA) models.

The results of the Mann-Whitney U Test comparison of FOCUSED and ARMA are shown in Table 3.

Input vectors are expressed the same way as in the statistical evaluation.

Finally, we rejected the null hypothesis because statistical significance of the algorithm’s performance in combination with all of tested data-mining models was confirmed by p-values lower than 0.05. The rejection of the null hypothesis and the differences between U_1_ and U_2_ values indicate that errors of FOCUSED corrections are systematically ranked lower than FD_6_ forecasts and ARMA correction method applied to the last received forecast.

### 4.3. Empirical Evaluation

Here we used past hourly averaged data for five consecutive years (2011–2016) with the following parameter values:

*T_s_* = 12 h, *F_p_* = 72 h, *T_p_* = 6 h

To measure the difference between the original and corrected forecasts, we used RMSE (Root Mean Square Error):

(17) $$RMSE=\sqrt{\frac{\sum_{i=1}^{n}{\left(v_{c}\left(t\right)-v_{m}\left(t\right)\right)}^{2}}{n}},$$

where n is the length of prediction, $v_{c}\left(t\right)$ is the corrected wind speed at time t, and $v_{m}\left(t\right)$ is the measured wind speed at time t.

To demonstrate the algorithm, we observed 53 wind situations considered dangerous for the road traffic over a period of five years. The modelling results for each sample are categorized according to the calculated RMSE values, as shown in Table 4.

**Figure 10:**
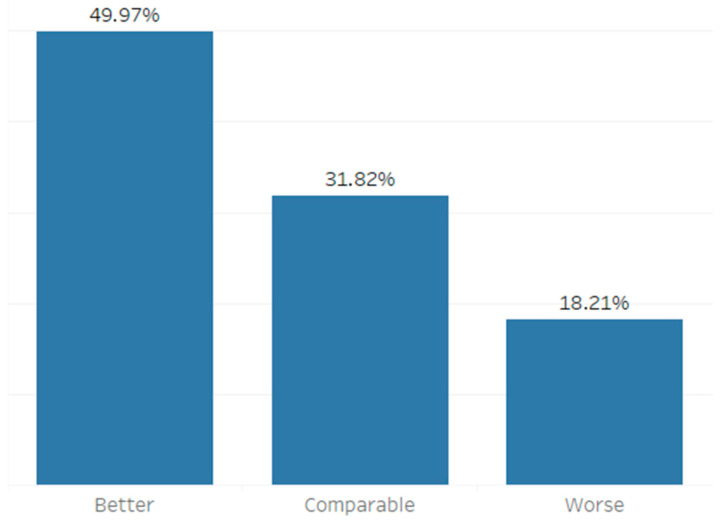
Distribution of the results of the correction algorithm applied to a real five-year data set from Croatian bridge Krk.

The granularity of the time series was one hour. The number of respective samples per strong wind situation varied depending on its duration, resulting in total of 1691 modelling samples (hours) distributed as shown in Figure 10.

**Figure 11:**
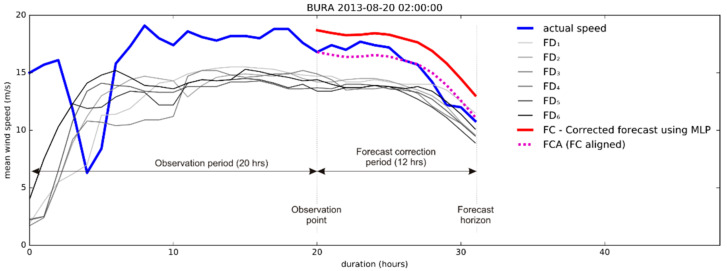
An example of the corrected 12 h forecast.

Figure 11 shows an example of the “Better” category sample. We present the first 20 h of six consecutive forecasts annotated with grey lines and increasing numbers. These forecasts are the source for the ANN. The oldest forecast is marked as FD_1_ and presented with the light grey line. It refers to the forecast created 72 h ago. The next one, created 60 h ago, is marked FD_2_ and is a bit darker than FD_1_. The last (most shaded grey) forecast, marked as FD_6_, represents the newest result of the ALADIN model. FC represents the direct output of the ANN model. A challenge has arisen as the prediction of the model revealed the most probable shape of the final forecast, but the overall wind speed was often shifted across ordinate of the graph, causing increased RMSE. To correct that, we aligned the curve’s wind speed starting point with the last measured data point. The FCA represents the aligned (final) corrected forecast.

**Figure 12:**
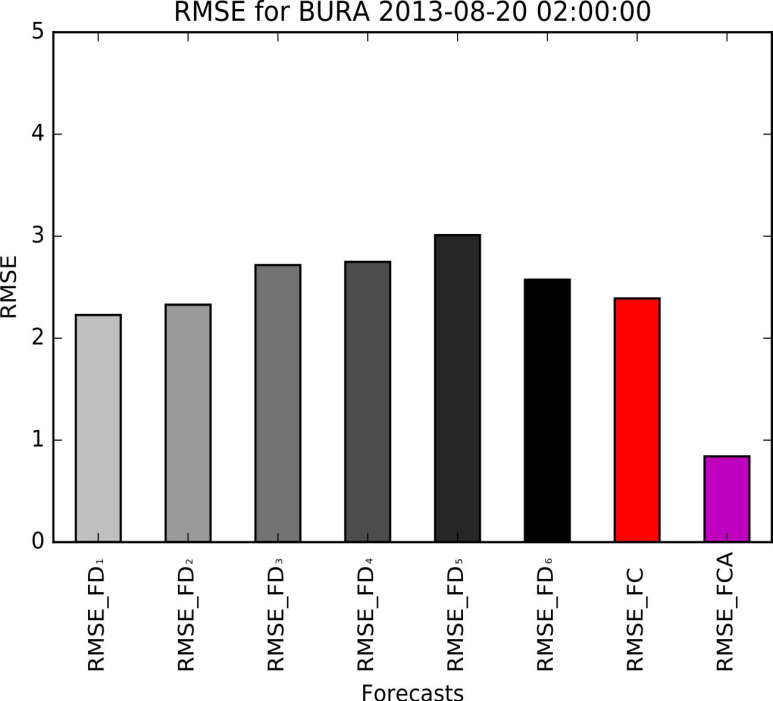
An example of improved accuracy: the final correction (RMSE_FCA) has better accuracy than any of the previous forecasts.

Figure 11 and Figure 12 show that FCA follows the actual speed curve more tightly than any of the previous FD_1_–FD_6_ forecasts.

Consequently, the algorithm outperformed the last ALADIN forecast as expected. The improvement of RMSE in the above example is confirmed trough comparison of actual speed with corrected forecast (Figure 12), showing that the result of the proposed algorithm surpasses the accuracy of FD_6_ (and, in this case, all previous forecasts).

This example is also interesting because the oldest prediction (72 h old, denoted as RMSE_FD1) is more accurate than the newest one (denoted as RMSE_FD6). The comparison of RMSE in the example of bora (Figure 11) shows the strength of the proposed algorithm to reduce the overall error of original forecasts, regardless of their order.

**Figure 13:**
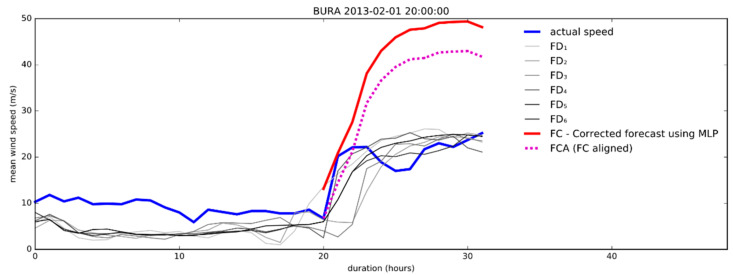
An example of the “Worse” category of the corrected forecast at the moment of a sudden large speed jump.

Figure 13 shows an example of the “Worse” category. Such significant discrepancies sometimes occur during the first hours after a sudden large jump of measured values in a short time, or when a strong wind situation occurs immediately after a long period of constant slow wind speed. Shortly after that sudden wind speed change (typically 1-3 h), results of the correction algorithm return to the “Better” or “Comparable” category again. We expect to minimize these discrepancies in further research by comparing forecasted wind speed curves shape with actual wind speed curve shape and minimizing the shape difference errors using time series transformations available in the REFII (Raise-Equal-Fall model Version II) methodology for holistic time series analysis, based on a time series transformation model. [27,28].

**Figure 14:**
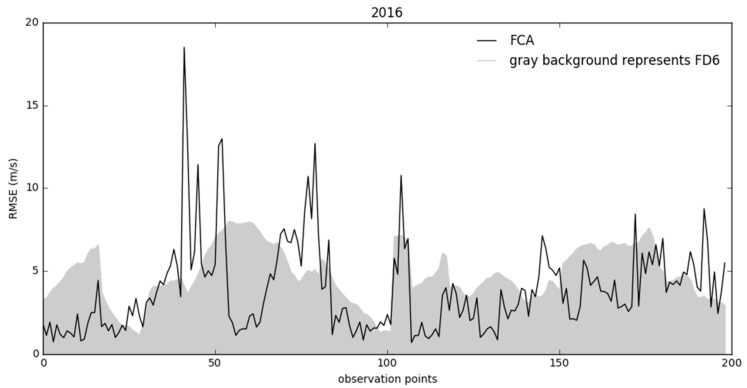
Comparison of hourly RMSE between corrected and uncorrected forecasts for one year. A reliable algorithm should fit the FCA curve inside FD_6_ (grey) area most of the time.

When we visualize the last NWP forecast (FD_6_) and the corrected forecast (FCA) during a longer period, we can spot better prevailing accuracy of FCA for one year. Figure 14 shows an example for year 2016—the comparison of FD_6_ (the newest and considered the most relevant before correction) and FCA for all training samples longer than or equal to 12 h. Comparison is made for all observation points (hours) in the whole data set, using only data from the 12th hour after the bora start (minimal training sample size is 12). The figure also shows relatively rare “Worse” situations, represented as high peaks on the graph. Those periods are typically short and followed by fast stabilization, resulting in further improvement of the model’s accuracy.

**Figure 15:**
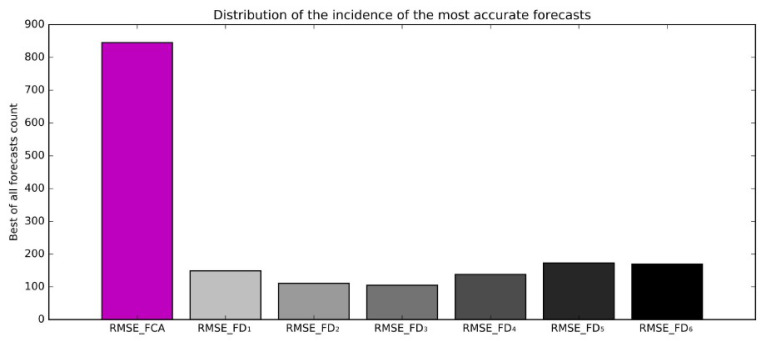
In the far majority of cases, FCA is the best compared to successive ALADIN forecasts.

The comparison of the results achieved with the FD_1_–FD_6_ and FCA forecasts shows that the FCA outperforms all original forecasts (FD_1_-FD_6_), as presented in Figure 15.

## 5. Conclusions and Future Work

In this paper, we present a short-term wind speed forecast correction algorithm. It uses previous successive forecasts as input features to an ANN and outputs the corrected value. We have shown that our algorithm outperforms all of the successive original forecasts in the majority of cases in a total of 1691 observed samples. In 49.97% of cases, our algorithm outperformed all of the original forecasts. In 31.82% of cases, the results were inside the range of the original forecasts. This research also shows that the newest forecast created with the NWP model is not always the best one in comparison with forecasts generated previously by the same model for the same prediction interval of 12 h, even though in practice it is used as if it were.

However, empirical evaluation of the algorithm also exposed significant degradations of the forecast accuracy in 18.21% cases, mostly after an abrupt change of measured values or when strong gusts occur immediately after a long period of relatively low wind speed. Since the correction of multiple previous forecasts for a specific time may depend on the congruence between the shape of the forecasted and measured data, we expect to solve these exceptional cases in further research by combining various segments of the time series transformation model REFII. As part of the future work, the presented algorithm will be further improved by adding non-meteorological input parameters, avoiding utilization of any input variables used during the creation of the original series of forecasts in order to keep the correction as non-biased as possible.

## Figures and Tables

**Table 1:** Training and test set samples for four algorithms’ runs.

Algorithm Run Number	Training Set Samples	Test Samples
1	1–12	13–24
2	1–13	14–25
3	1–14	15–26
4	1–15	16–27

**Table 2:** Results of Mann-Whitney U Test.

Tested Model	*p*-Value	U_1_-FOCUSED	U_2_-FD_6_
FCA_ANN	0.0015	109,164	135,861
FCA_RFR	0.0017	109,366	135,659
FCA_SVM	0.0322	114,195	130,830
FCA_LRM	0.0348	114,348	130,677

**Table 3:** Results of Mann-Whitney U test comparison of FOCUSED and ARMA models.

Tested Algorithm	*p*-Value	U_1_-FOCUSED	U_2_-ARMA
FOCUSED	0.0015	109,164	135,861
ARMA	0.0139	113,076	132,940

**Table 4:** Algorithm’s performance categories.

Category	Description
Better	RMSE FCA lower than any RMSE of original NWP forecasts
Comparable	RMSE FCA between lowest and highest RMSE of original NWP forecasts
Worse	RMSE FCA higher than any RMSE of original NWP forecasts

## Data Availability

The data that support the findings of this study are available from Croatian Meteorological and Hydrological Service. Restrictions apply to the availability of these data, which were used under license for this study. The procedure and request form to access weather data are available at https://meteo.hr/proizvodi_e.php?section=proizvodi_usluge&param=services, accessed on 31 March 2021.
